# Survival of Surfactant Protein-A1 and SP-A2 Transgenic Mice After *Klebsiella pneumoniae* Infection, Exhibits Sex-, Gene-, and Variant Specific Differences; Treatment With Surfactant Protein Improves Survival

**DOI:** 10.3389/fimmu.2018.02404

**Published:** 2018-10-16

**Authors:** Nithyananda Thorenoor, Todd M. Umstead, Xuesheng Zhang, David S. Phelps, Joanna Floros

**Affiliations:** ^1^Center for Host defense, Inflammation, and Lung Disease (CHILD) Research, Department of Pediatrics, The Pennsylvania State University College of Medicine, Hershey, PA, United States; ^2^Department of Obstetrics & Gynecology, The Pennsylvania State University College of Medicine, Hershey, PA, United States

**Keywords:** pulmonary surfactant protein A, pneumonia infection, surfactant protein A1 and A2, sex differences, innate host defense

## Abstract

Surfactant protein A (SP-A) is involved in lung innate host defense and surfactant-related functions. The human SFTPA1 and SFTPA2 genes encode SP-A1 and SP-2 proteins, and each gene has been identified with numerous genetic variants. SP-A1 and SP-A2 differentially enhance bacterial phagocytosis. Sex differences have been observed in pulmonary disease and in survival of wild type and SP-A knockout (KO) mice. The impact of human SP-A variants on survival after infection is unknown. In this study, we determined whether SP-A variants differentially affect survival of male and female mice infected with *Klebsiella pneumoniae*. Transgenic (TG) mice, where each carries a different human (h) SP-A1 (6A^2^, 6A^4^), SP-A2 (1A^0^, 1A^3^) variant or both variants SP-A1/SP-A2 (6A^2^/1A^0^, co-ex), and SP-A- KO, were utilized. The hTG and KO mice were infected intratracheally with *K. pneumoniae* bacteria, and groups of KO mice were treated with SP-A1 or SP-A2 either prior to and/or at the time of infection and survival for both experimental groups was monitored over 14 days. The binding of purified SP-A1 and SP-A2 proteins to phagocytic and non-phagocytic cells and expression of cell surface proteins in alveolar macrophages (AM) from SP-A1 and SP-A2 mice was examined. We observed gene-, variant-, and sex-specific (except for co-ex) differences with females showing better survival: (a) Gene-specific differences: co-ex = SP-A2 > SP-A1 > KO (both sexes); (b) Variant-specific survival co-ex (6A^2^/1A^0^) = 1A^0^ > 1A^3^ = 6A^2^ > 6A^4^ (both sexes); (c) KO mice treated with SPs (SP-A1 or SP-A2) proteins exhibit significantly (*p* < 0.05) better survival; (d) SP-A1 and SP-A2 differentially bind to phagocytic, but not to non-phagocytic cells, and AM from SP-A1 and SP-A2 hTG mice exhibit differential expression of cell surface proteins. Our results indicate that sex and SP-A genetics differentially affect survival after infection and that exogenous SP-A1/SP-A2 treatment significantly improves survival. We postulate that the differential SP-A1/SP-A2 binding to the phagocytic cells and the differential expression of cell surface proteins that bind SP-A by AM from SP-A1 and SP-A2 mice play a role in this process. These findings provide insight into the importance of sex and innate immunity genetics in survival following infection.

## Introduction

Cells and molecules of innate immunity in the lung provide the first line of host defense against a number of harmful pathogens, allergens, and air pollutants. The major outcomes of lung infection depend on several factors, such as effective clearance of pathogens and extra-pulmonary dissemination of infection into other internal organs. Pneumonia is a major health problem and a significant cause of infectious disease-related illness and death (1, 2). It has been observed previously in animal studies that, during pneumonia infection, pulmonary clearance, and the ability to limit bacterial dissemination, play an important role in differential outcome in survival between males and females in the presence or absence of oxidative stress (3, 4).

The Gram-negative bacteria *Klebsiella pneumoniae* (*K. pneumoniae*) are the most common hospital-acquired pathogens of the respiratory tract (5, 6). *K. pneumoniae* causes a wide range of infections, including pneumonia, urinary tract infections, bacteremias, and liver abscesses. Although immunocompromised individuals are more affected by *K. pneumoniae* infection, the development of new hypervirulent strains has affected a number of people including those who are healthy and immunosufficient or immunocompetent (7). The spread of *K. pneumoniae* infection to the blood stream is a critical step in disease pathogenesis, which leads to multiple organ failure (8).

Surfactant protein A (SP-A) is an important innate immune defense molecule. The human SP-A genetic locus consists of two functional genes, SFTPA1 and SFTPA2, encoding human SP-A1 and SP-A2, respectively. Each has been identified with a number of variants (9, 10). The SP-A1 and SP-A2 variants have been identified with both qualitative (i.e., functional, biochemical and/or structural) (11–23), and quantitative (regulatory) differences (24–28). The SP-A1 and SP-A2 variants have been shown to differ in their ability to modulate the proteomic expression profile of AM and the AM actin cytoskeleton (29–31), the AM miRNome (32), and the biophysical function of surfactant, where SP-A1 exhibits a higher efficiency in pulmonary surfactant reorganization (33). However, no differences have been observed between SP-A1 (6A^2^) and SP-A2 (1A^0^) in the inhibition of hemagglutination activity of influenza virus when *in vitro* expressed SP-A1 and SP-A2 was used (34).

SP-A has been found to enhance phagocytosis of microspheres, by exerting a direct effect on the phagocytic cell itself (35). Previously, we have also shown that SP-A2 variants enhance bacterial cell association and phagocytosis more effectively than SP-A1 variants (18, 19), and this activity is differentially compromised in response to oxidative stress (22). Furthermore, sex-dependent survival was observed in wild type and SP-A KO mice in response to *K. pneumoniae* infection, with females exhibiting higher survival compared to males, and the reverse was observed after oxidative stress (4, 36, 37), with females exhibiting lower survival compared to males. Sex hormones were implicated in the differential survival (38). Recent findings indicated that the SP-A1 and SP-A2 variants play a crucial role in the differential outcome of airway function in males and females with significant sex- and gene-specific differences in airway function mechanics in response to *K. pneumoniae* infection and methacholine challenge (23).

In the present study building on our previous findings we investigated the role of two SP-A1 (6A^2^, 6A^4^) and two SP-A2 (1A^0^, 1A^3^) variants, which are frequently observed in the general population (10), on survival in response to *K. pneumoniae*. We also studied whether treatment with SP-A1 or SP-A2 prior to and/or at the time of infection improves survival. In an attempt to gain insight into potential mechanisms, we studied SP-A1 and SP-A2 binding to phagocytic cells and differential expression AM cell surface proteins in SP-A1 and SP-A2 mice. The findings indicated a differential outcome in survival that is sex-, gene-, and variant- specific, and differential binding and differential expression of cell surface proteins may contribute to the observed differences.

## Methods

### Animals

We used humanized transgenic (hTG) mice that each carried SP-A1 (6A^2^, 6A^4^), SP-A2 (1A^0^, 1A^3^), or both variants SP-A1/SP-A2 (6A^2^/1A^0^, co-ex), as well as SP-A knockout (KO) mice. hTG mice were generated on the C57BL6/J SP-A (KO) background (39). The animals used in this study were maintained as described previously (23). Briefly, the animals were raised and maintained in a pathogen-free environment, at the Penn State College of Medicine animal facility. Both males and females were used in this study. The females were synchronized with regard to the estrous cycle by placing female (group housed) mice in a dirty bedding from male cages 7 days prior to infection to stimulate estrus. All mice used in the present study were ~12 weeks of age. A total of 490 mice (484 for infection and survival, 6 for Flow cytometry analysis) and 3 Sprague Dawley male rats (Harlan, Indianapolis, IN) for binding assay were used in the present study. All the procedures involving animal studies were approved by The Penn State Hershey Medical Center Institutional Animal Care and Use Committee (IACUC).

### Preparation of bacteria

*K. pneumoniae* bacteria (ATCC 43816) were obtained from American Tissue Culture Collection (Rockville, MD) and prepared as described previously (23, 37). Fifty μl of a suspension containing ~ 450 CFU was used to infect each mouse. CFU/ml values were calculated based on the standard curve obtained at OD_660_ of the bacterial suspension.

### Infection of mice with *K. pneumoniae*

Mice were infected and monitored as described previously (23, 37). Briefly, hTG mice, SP-A1 (6A^2^, 6A^4^), SP-A2 (1A^0^, 1A^3^), SP-A1/SP-A2 (6A^2^/1A^0^, co-ex), and SP-A KO male and female mice were anesthetized, and infected intratracheally with *K. pneumoniae*, and monitored for survival twice a day for 14 days. Mice fallen sick and unable to recover, were euthanized immediately according to Penn State University IACUC protocol.

### Treatment of mice with SP-A protein

SP-A KO male and female mice were anesthetized and pretreated with vehicle (0.9% NaCl, and 1 mM CaCl_2_) and/or 10 μg of SP-A1 (6A^2^), SP-A2 (1A^0^) protein in 50 μl of 0.9% NaCl, and 1 mM CaCl_2_ intratracheally for 18 h prior to infection. The mice were infected with *K. pneumoniae* (~450 CFU/mouse) as described above, in combination with vehicle or with 10 μg of purified SP-A1 or SP-A2 protein expressed by stably transfected CHO cells as described (13, 17) in 50 μl of PBS intratracheally (23, 37), and monitored for survival for 14 days.

### SP-A1 and SP-A2 variant binding assay

Bronchoalveolar lavage was performed using PBS containing 1mM EDTA. AMs were then washed two times with PBS, 1mM EDTA and re-suspended in HBSS and adhered to 96-well, flat-bottom tissue culture plates. Adherent cells were washed with HBSS and blocked with HBSS containing 1 mg/mL human serum albumin. Plates were washed with binding buffer (10 mM HEPES/NaOH (pH 7.4), 0.14 M sodium chloride, 2.5 mM calcium chloride) and then incubated with 2.5 μg/mL of SP-A2 or SP-A1 in binding buffer. After binding, plates were washed four times with binding buffer and AM were extracted with extraction buffer (Tris-buffered saline, 6% SDS, 1% β-mercaptoethanol, 1mM EDTA). The extracted AMs were then diluted in TBS, and filtered using a Pall AcroPrep 96 filter plate to remove particulates. The samples were analyzed by dot blot method using nitrocellulose membrane and SP-A protein binding was detected using rabbit polyclonal antibody against hSP-A (1:25,000) and a goat anti–rabbit (IgG) HRP-conjugated (1:25,000, Bio-rad) and quantified by densitometry. For binding assays with THP-1(human monocyte derived, a macrophage-like and phagocytic) cell line, and CHO (non-phagocytic), cells were cultured as previously described (11, 13, 17), and assays were performed as mentioned above for AM cells.

### Measurement of AM cell surface proteins by flow cytometry

Staining for flow cytometry was performed on mouse alveolar macrophages (AMs) obtained from bronchoalveolar lavage of SP-A1 (6A^2^) and SP-A2 (1A^0^) hTG mice. Cells were washed with FACS buffer (PBS, 0.1 mM EDTA, 1% BSA, 0.02% sodium azide) and then blocked for 20 min at 4°C in FACS buffer containing 2 μg/mL Mouse BD Fc Block (BD Biosciences, Franklin Lakes, NJ) and 5% normal mouse serum. Fluorescently-conjugated mouse antibodies (PE-labeled) to specific cell surface markers (CD14 and TLR2, eBioscience, NY) were then added (1 μL/reaction) directly to the blocking solution and incubated for 30 min at room temperature. Cells were next washed 2 times with FACS buffer and fixed for 30 min at 4°C with 1% paraformaldehyde in FACS buffer. AMs were washed and re-suspended in FACS buffer and analyzed using a Special Order BD LSR II flow cytometer (BD). AMs were gated on the basis of forward scatter and side scatter and data for the expression of fluorescently-labeled cell surface proteins were collected from a population of 1 × 10^4^ gated AMs using FACS Diva (BD) and analyzed using Cyflogic (CyFlo Ltd., Turku, Finland).

### Statistical analysis

Survival was analyzed by Chi-Square test (daily survival). The surviving animals were compared with a one-way analysis of variance (ANOVA) followed by Bonferroni multiple comparisons correction for each experimental group. Two-tailed *t*-tests were used to compare the SP-A variant binding assay results, and cell surface protein measurements. Data were presented as ± standard deviation. *P*-value < 0.05 was considered to be significant (GraphPad Prism version 5; GraphPad Software, San Diego, USA).

## Results

hTG mice, SP-A1 (6A^2^, 6A^4^), SP-A2 (1A^0^, 1A^3^), SP-A1/SP-A2 (6A^2^/1A^0^, co-ex), and SP-A KO male and female mice were infected with *K. pneumoniae*, and monitored for 14 days for survival.

**Figure 1:**
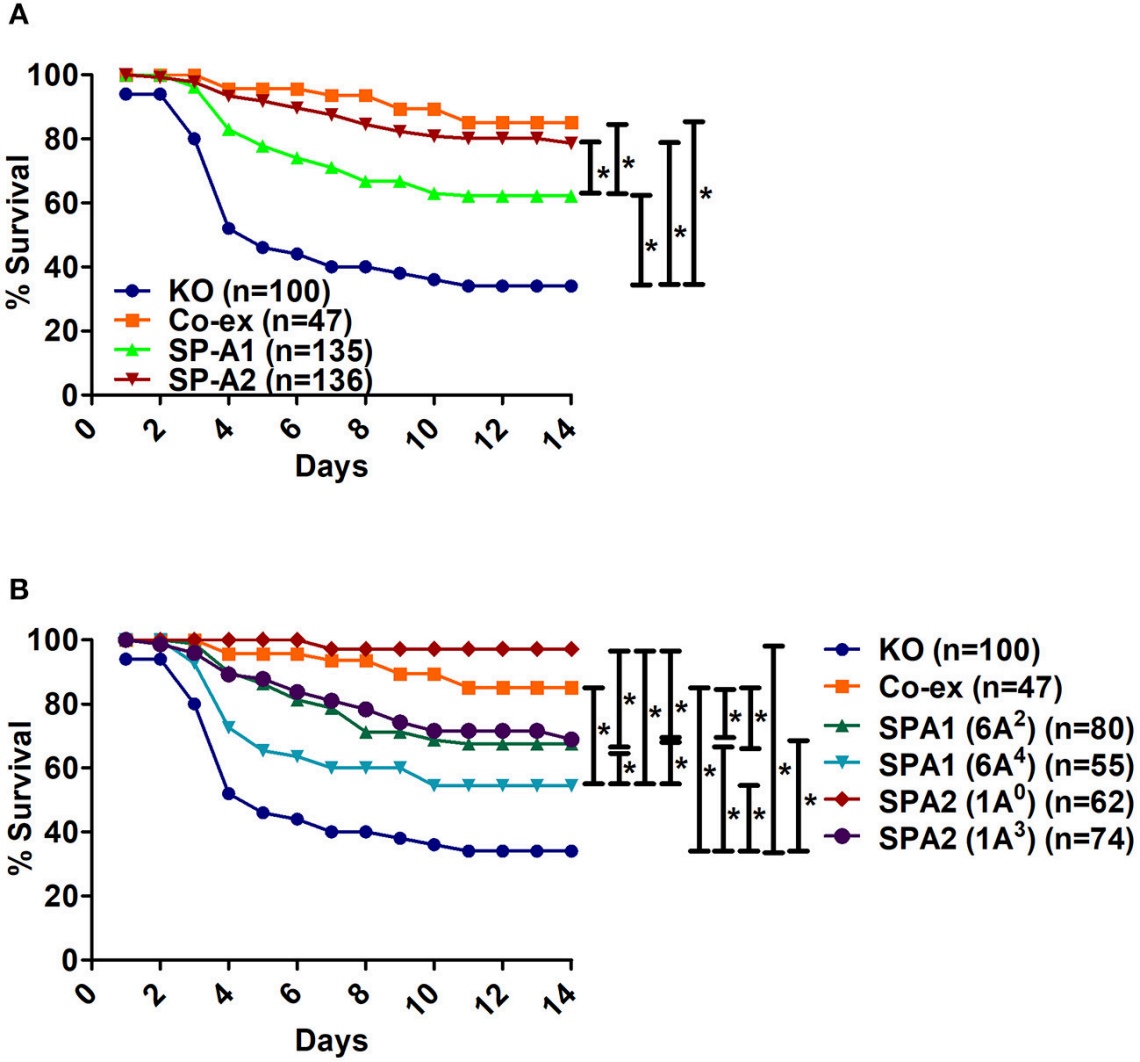
Comparison of survival after *K. pneumoniae* infection. **(A)** Depicts differences in survival of male and female mice carrying a single SP-A1 or SP-A2 variant or both SP-A1 and SP-A2 (co-ex) variants and lacking SP-A (KO). **(B)** Depicts differences in survival of KO, SP-A2 (1A^0^, 1A^3^), SP-A1 (6A^2^, 6A^4^) and co-ex (males and females combined). Significant differences are indicated for survival ^*^p < 0.05.

When we compared the overall survival of SP-A1, SP-A2, and mice carrying both SP-A variants (co-ex) we found that all mice had a better survival than the KO indicating that SP-A protects them from *K.pneumoniae* infection. SP-A2 and co-ex mice exhibited similar survival rates and better than mice carrying SP-A1 variants (Figure 1A). Thus the survival is genotype specific in the following order, co-ex = SP-A2 > SP-A1 > KO.

Next, we investigated whether survival differences exist between variants of a given gene or among variants. The survival (males and females combined) of SP-A variants (1A^0^, 1A^3^, 6A^2^, 6A^4^), co-ex, and KO, is shown in Figure 1B. Mice carrying either an SP-A single gene variant or both gene variants (co-ex) had significantly higher survival compared to KO. Co-ex and SP-A2 (1A^0^) had a similar survival rate over a 14 day period and this was significantly (*p* < 0.05) higher than that observed for mice carrying SP-A1 (6A^2^, 6A^4^) and SP-A2 (1A^3^) variants, indicating a gene and variant specific survival (Figure 1B). The mice carrying the SP-A2 (1A^3^) variant had similar survival rate with those carrying the SP-A1 (6A^2^), and both exhibited significantly better survival compared to 6A^4^ variant (Figure 1B). The order of survival is, coex = SP-A2 (1A^0^) > SP-A2 (1A^3^) = SP-A1 (6A^2^) > SP-A1 (6A^4^) > KO.

### Effect of sex and SP-A variants on the course of pneumonia

#### Sex differences in survival of between SP-A1, SP-A2, KO, and SP-A1/SP-A2 (6A^2^/1A^0^, co-ex) mice

**Figure 2:**
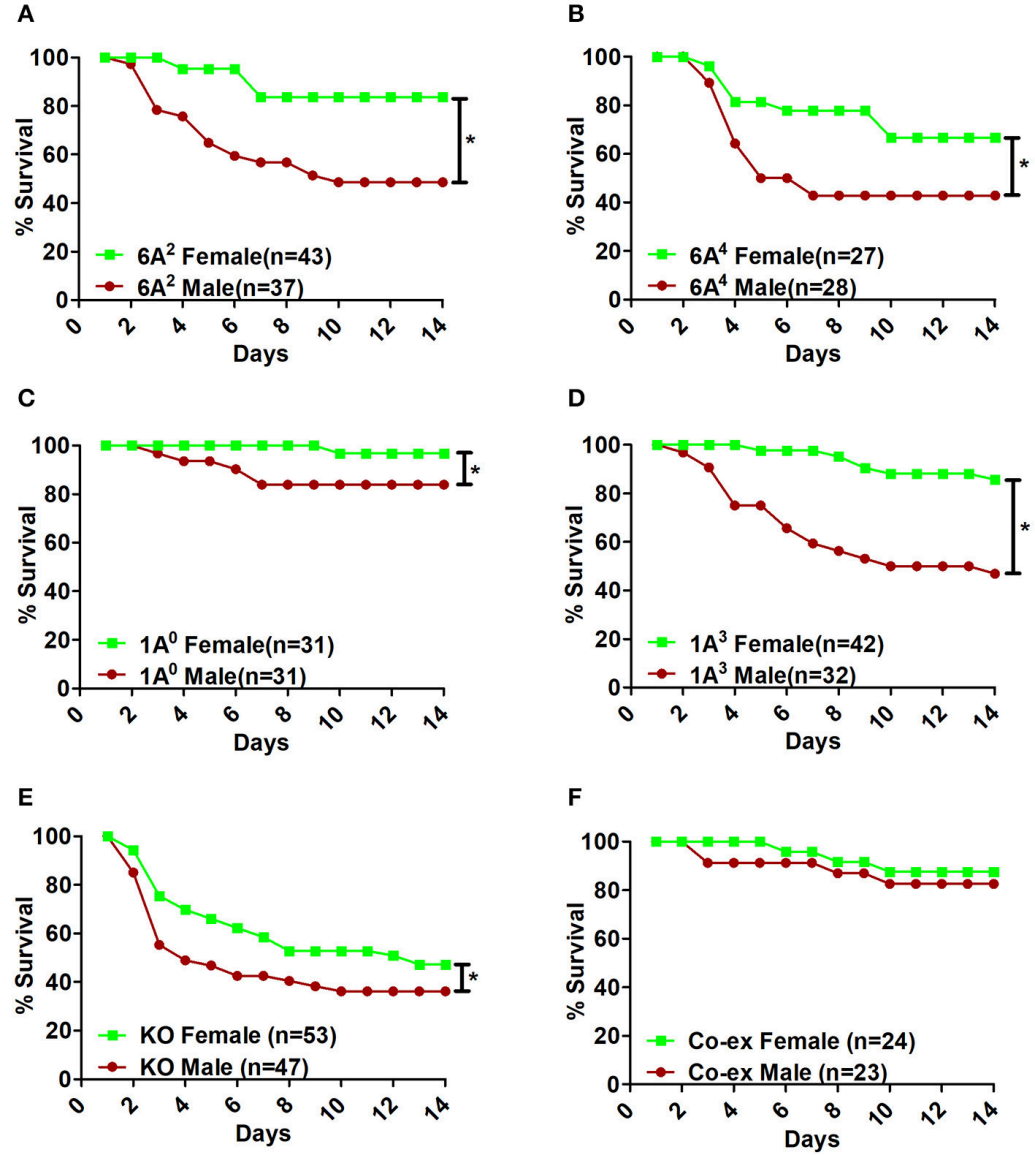
Effect of sex on survival after *K. pneumoniae* infection. The survival rate was measured in SP-A1 (6A^2^, 6A^4^) **(A,B)**, SP-A2 (1A^0^, 1A^3^) **(C,D)**, SP-A KO **(E)**, and SP-A1/SP-A2 [6A2/1A^0^, (co-ex)] **(F)** male and female mice over a period of 14 days after infection. Significant differences for survival are indicated ^*^p < 0.05. The number of mice used in each group is shown in Figure panels in parenthesis (*n* =).

Infection with *K.pneumoniae* resulted in significant sex differences over a period of time. In the SP-A1 (6A^2^, 6A^4^), SP-A2 (1A^0^, 1A^3^), and KO groups, all males compared to females showed a significant decrease in survival after infection (Figures 2A–E). The difference seemed to be considerably larger between males and females for 6A^2^ and 1A^3^ mice and to a lesser degree for 6A^4^ mice (Figures 2A,B,D), whereas, 1A^0^ and KO showed small but significant difference between males and females (Figures 2C,E). However, in mice carrying both SP-A1/SP-A2 variants (6A^2^/1A^0^, co-ex), no significant sex differences in survival were observed (Figure 2F).

#### Sex differences between gene-specific variants

##### SP-A1

**Figure 3:**
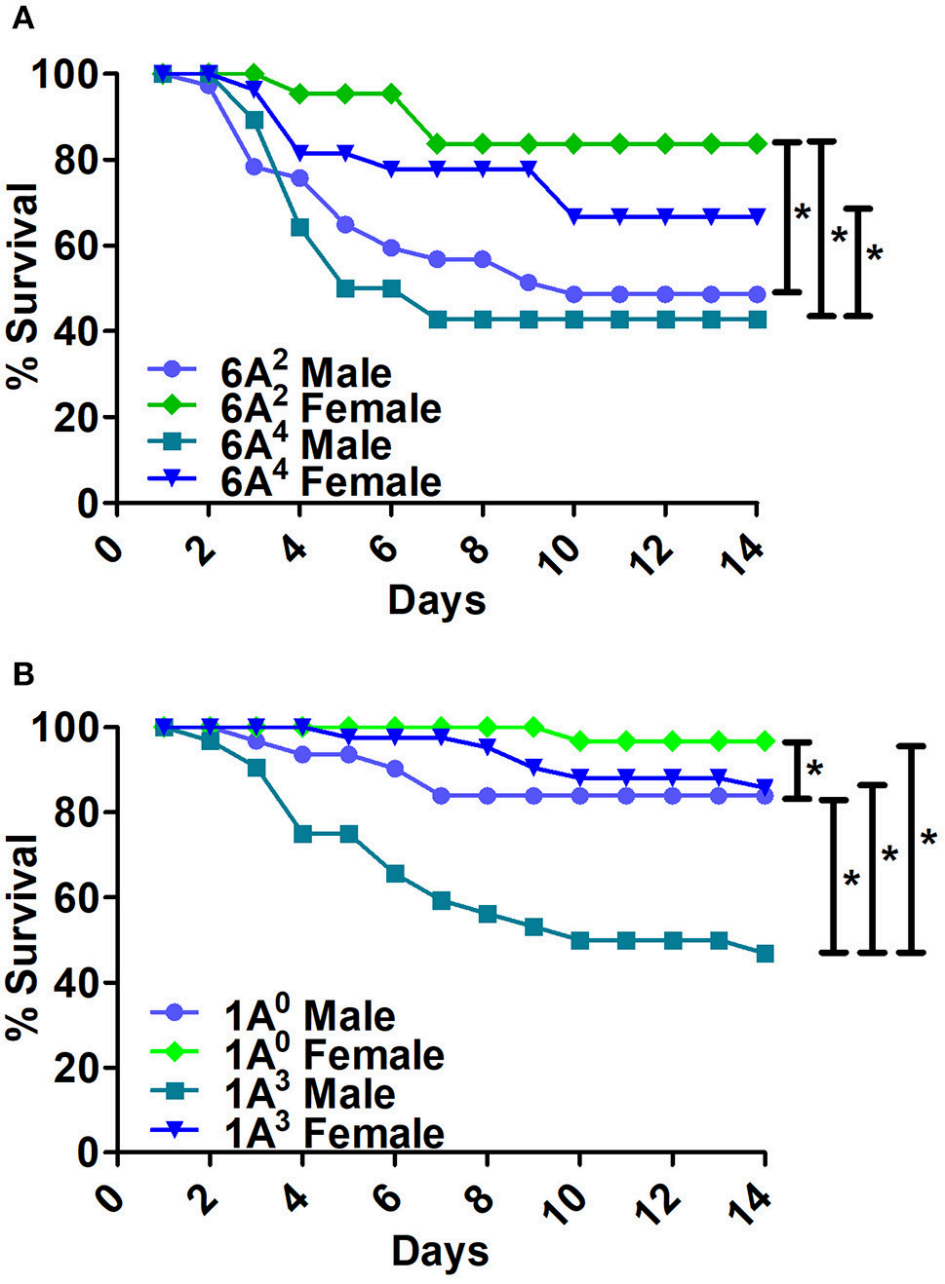
Comparison of survival as a function of SP-A gene-specific variant after *K. pneumoniae* infection**. (A)** Depicts differences in survival between male and female mice of SP-A1 (6A^2^, 6A^4^) and **(B)** depicts differences of SP-A2 (1A^0^, 1A^3^) variants. Mouse survival was monitored for 14 days after infection. Significant differences for survival are indicated ^*^p < 0.05. The number of mice used for each group is shown in Figure 2.

Bacterial infection resulted in significant differences in survival between males and females of 6A^2^ and 6A^4^ variants (Figure 3A). The 6A^2^ and 6A^4^ females had significantly higher survival compared to their respective 6A^2^ and 6A^4^ males, whereas, no significant differences were observed in males or females between each genotype (Figure 3A).

##### SP-A2

The 1A^0^ and 1A^3^ females had higher survival compared to their respective males (Figure 3B). 1A^0^ male had significantly higher survival compared to 1A^3^ males but similar to 1A^3^ females. Whereas, the 1A^0^ female had significantly higher survival compared to 1A^3^ male (Figure 3B).

#### Differences among SP-A1 and SP-A2 variants

**Figure 4:**
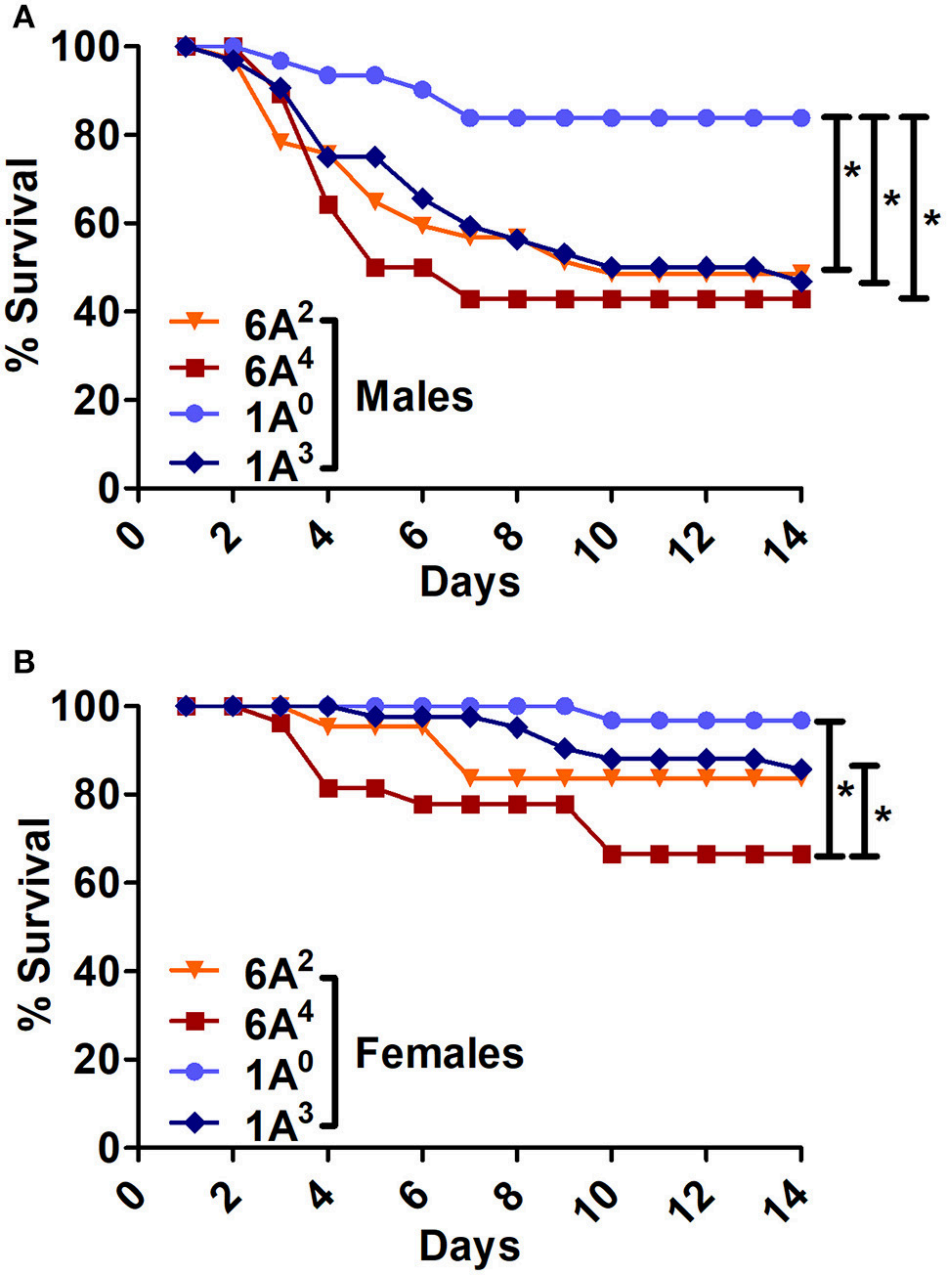
Comparison of survival among SP-A1 and SP-A2 variants after *K. pneumoniae* infection. **(A)** Depicts differences in survival among males and **(B)** Depicts differences among females of SP-A1 (6A^2^, 6A^4^) and SP-A2 (1A^0^, 1A^3^). Mouse survival was monitored for 14 days after infection. Significant differences for survival are indicated ^*^p < 0.05. The number of mice used for each group is shown in Figure 2.

The SP-A2 (1A^0^) males exhibited significantly higher survival compared to SP-A1 (6A^2^, 6A^4^) and SP-A2 (1A^3^) males (Figure 4A). The 1A^0^ females exhibited a significant increase in survival compared to 6A^4^ females, but similar to 1A^3^ and 6A^2^ females (Figure 4B).

The SP-A2 (1A^3^) males exhibited similar survival compared to 6A^2^, 6A^4^ males but lower than 1A^0^ (Figure 4A), whereas 1A^3^ females had significantly higher survival compared to 6A^4^ females, and similar to 6A^2^ and 1A^0^ females (Figure 4B).

In summary, Males: SP-A2 (1A^0^) > SP-A2 (1A^3^) = SP-A1 (6A^2^, 6A^4^); Females: SP-A2 (1A^0^) = SP-A2 (1A^3^) = SP-A1 (6A^2^) > (6A^4^).

#### Differences between SP-A1/SP-A2 (co-ex) or KO and SP-A1 or SP-A2 variants:

**Figure 5:**
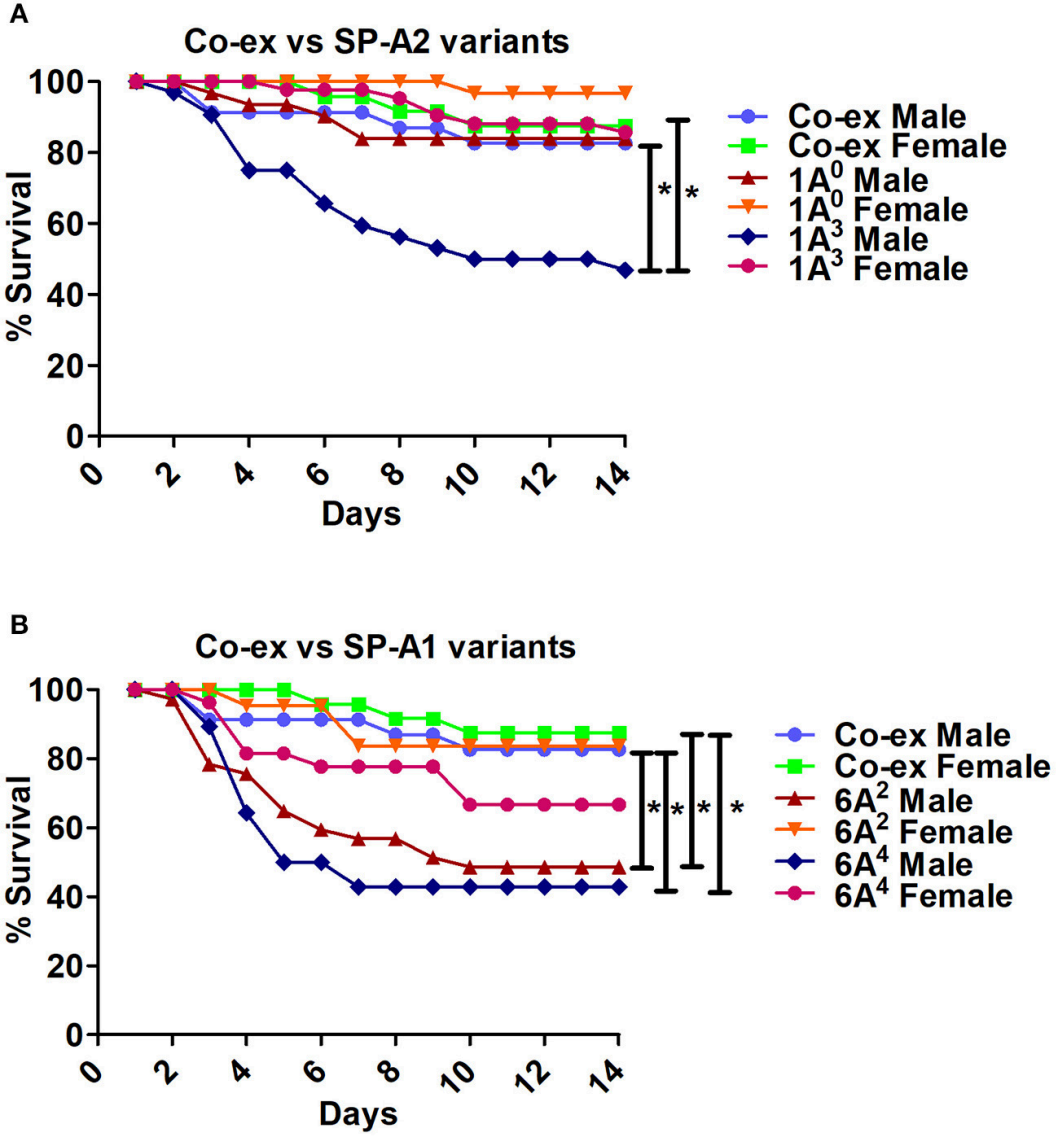
Comparison of survival between co-ex and single gene SP-A variants after *K. pneumoniae* infection**. (A)** Depicts differences in survival between males and females of co-ex vs. SP-A2 (1A^0^, 1A^3^) and **(B)** depicts differences of co-ex vs. SP-A1 (6A^2^, 6A^4^). Mouse survival was monitored for 14 days after infection. Significant differences for survival are indicated ^*^p < 0.05. The number of mice used for each group is shown in Figure 2.

**Figure 6:**
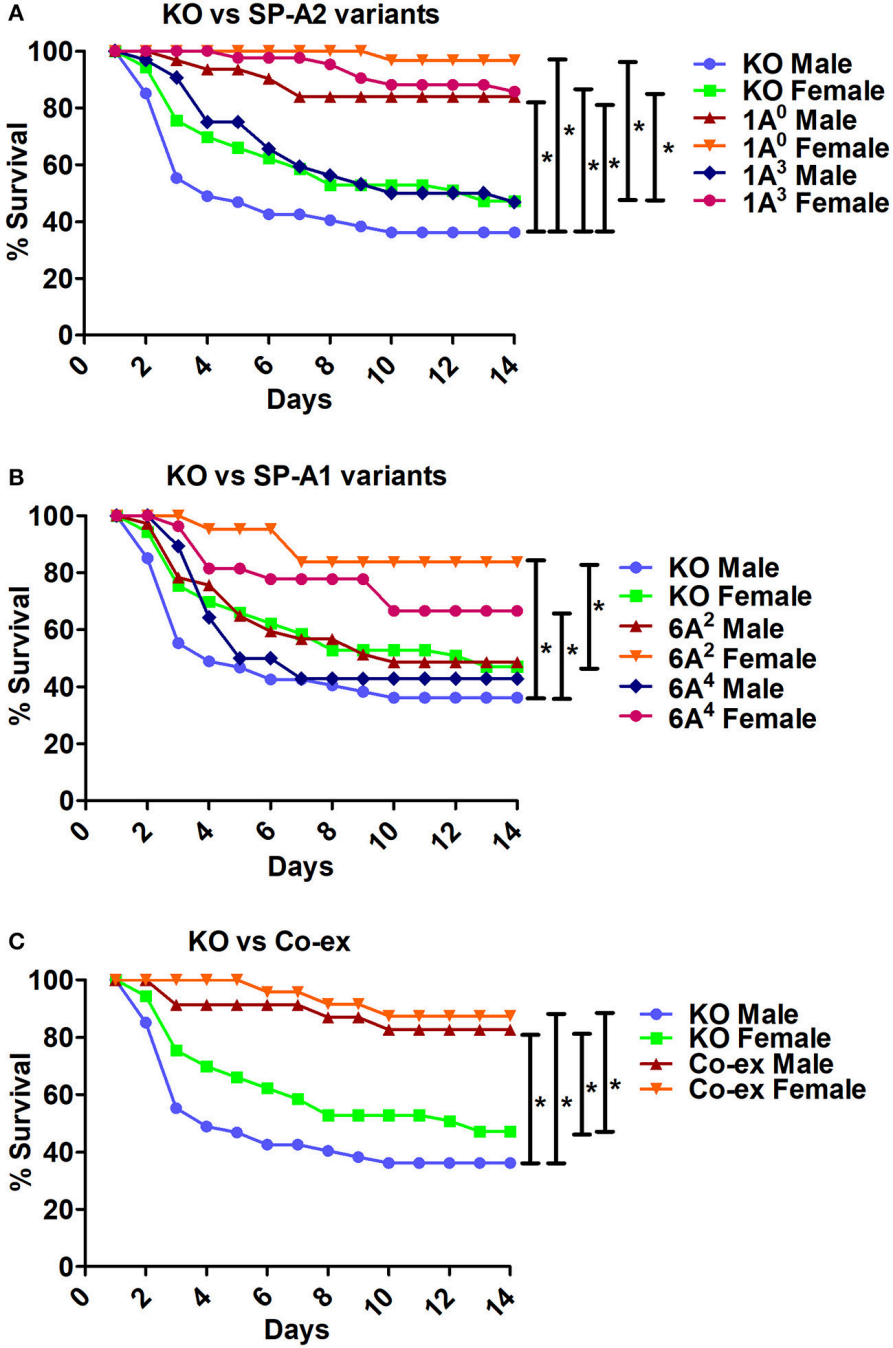
Comparison of survival between KO and SP-A variants, and between KO and co-ex after *K. pneumoniae* infection**. (A)** Depicts differences in survival between males and females of KO vs. SP-A2 (1A^0^, 1A^3^), **(B)** depicts differences between KO vs. SP-A1 (6A^2^, 6A^4^), and **(C)** depicts differences between KO vs. co-ex. Mouse survival was monitored for 14 days after infection. Significant differences for survival are indicated ^*^p < 0.05. The number of mice used for each group is shown in Figure 2.

- The SP-A1/SP-A2 (co-ex) exhibited significant differences in survival compared to SP-A single gene variants in both males and females, as shown in Figures 5A,B. The co-ex males and females exhibited significantly higher survival compared to 1A^3^ (Figure 5A) and 6A^2^, and 6A^4^ males (Figure 5B) males. Of interest, the survival rate of co-ex males and females was similar to 1A^0^ males and females, and 1A^3^ females (Figure 5A), and 6A^2^ and 6A^4^ females (Figure 5B).
- The KO exhibited significant differences in survival compared to SP-A variants and co-ex for both males and females, as shown in Figures 6A,B. The KO males and females had significantly lower survival compared to 1A^0^ males and females, 1A^3^ females (Figure 6A), 6A^2^ and 6A^4^ females (Figure 6B). However, the survival rate of KO males and females was similar to 1A^3^ males (Figure 6A), and 6A^2^ and 6A^4^ males (Figure 6B).
- The SP-A1/SP-A2 (co-ex) exhibited significant differences in survival compared to KO in both males and females, as shown in Figure 6C.

### Effect of exogenous SP-A1 and SP-A2 treatment on survival

**Figure 7:**
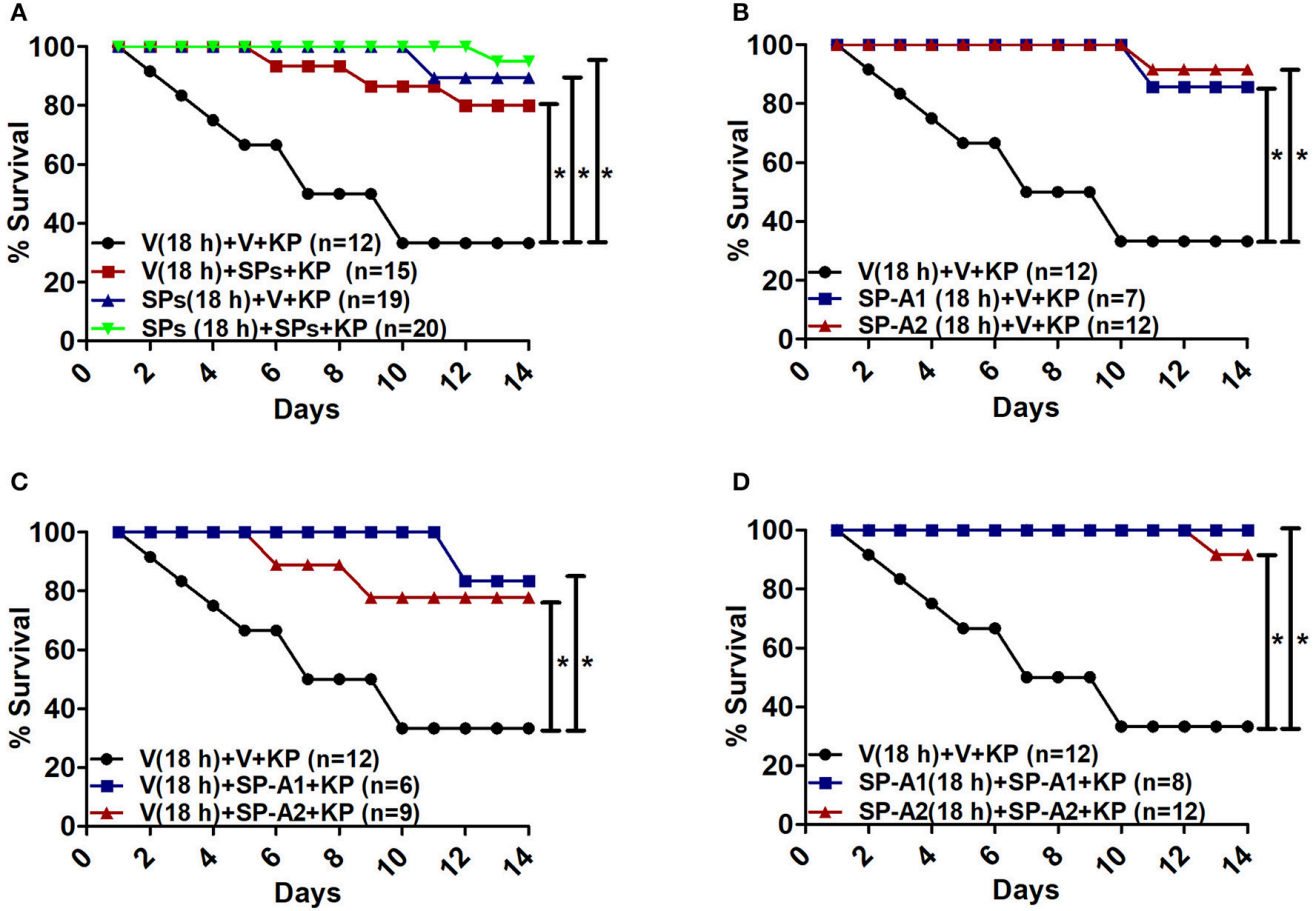
Rescue by exogenous SP-A1 or SP-A2 treatment**. (A)** Depicts overall survival of infected KO mice after surfactant protein (SPs) treatment. **(B)** Depicts KO mouse survival after pretreatment with SP-A1 or SP-A2 prior to infection, **(C)** depicts KO survival after pretreatment with vehicle (Vp) and rescue at the time of infection with SP-A1 or SP-A2, **(D)** depicts KO survival after pretreatment with SP-A1 or SP-A2 and rescue with SP-A1 or SP-A2 at the time of infection. Mouse survival was monitored for 14 days after infection and treatment. Significant differences are indicated for survival ^*^p < 0.05.

The overall survival of KO mice (male and female combined) that were treated prior to infection and/or at the time of infection with 10 μg of SP-A1 (6A^2^), or SP-A2 (1A^0^) purified proteins (indicated as SPs in Figure 7A) is shown in Figure 7A. SP-A treatment prior to infection, at the time of infection or prior to and at the time of infection resulted in a significantly better survival than mice treated with vehicle alone. However, no significant difference was observed among SP-treated groups (Figure 7A). No significant difference was observed (a) between vehicle and SP-A1 or SP-A2 treatment prior to infection (Figure 7B), (b) between vehicle and SP-A1 or SP-A2 treatment at the time of infection (Figure 7C), or (c) between vehicle and SP-A1 or SP-A2 treatment prior to infection and at the time of infection (Figure 7D).

### Binding of SP-A variants to phagocytic and non-phagocytic cells and cell surface protein expression by AM from SP-A1 and SP-A2 mice

**Figure 8:**
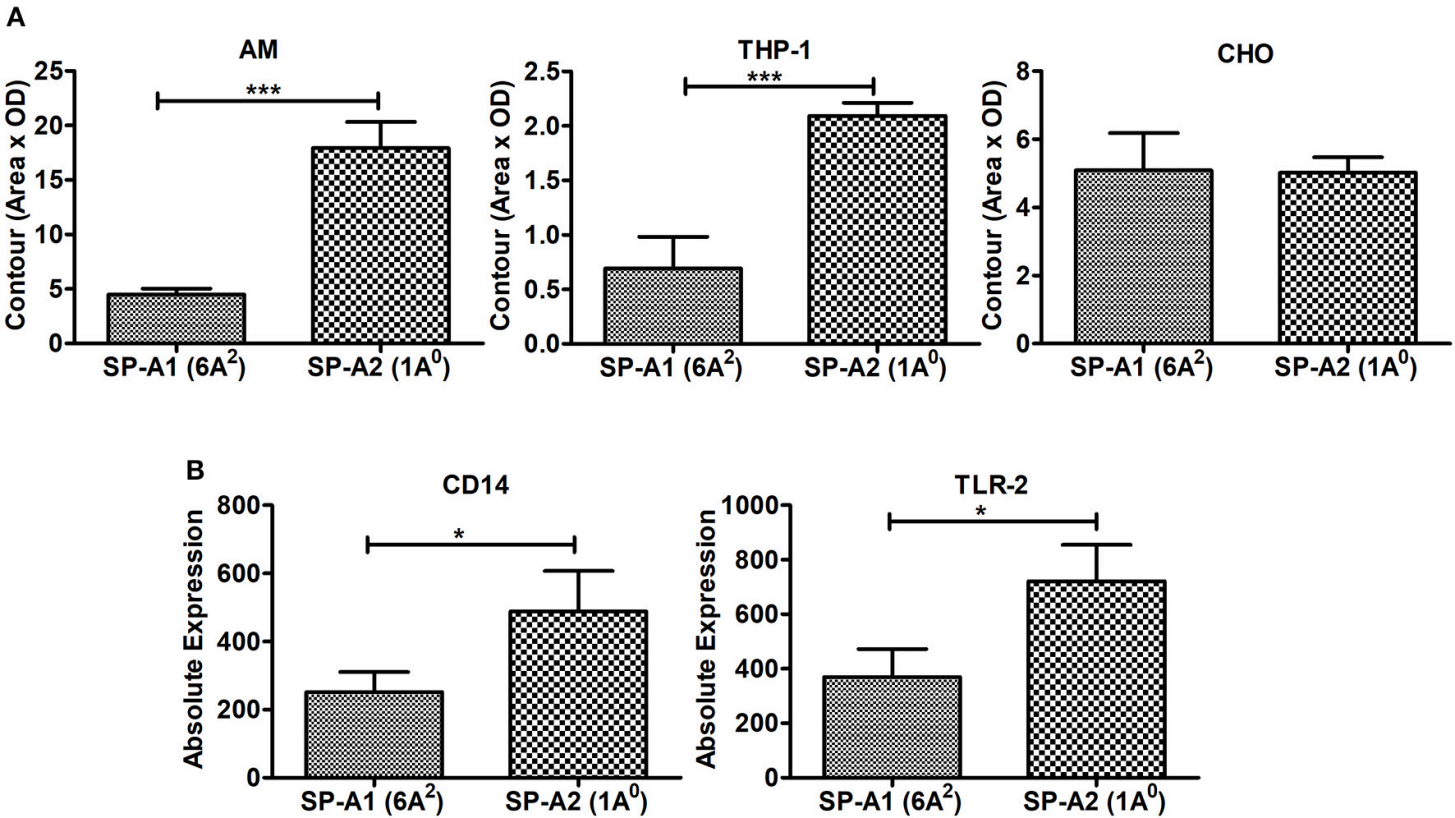
Binding of SP-A1 (6A^2^) and SP-A2 (1A^0^) variants to phagocytic and non-phagocytic cells and expression of cell surface proteins in AMs of SP-A1 (6A^2^) and SP-A2 (1A^0^) mice. **(A)** depicts binding of rat AM and two cell lines (THP-1, a macrophage-like and phagocytic and the other CHO, non-phagocytic) to SP-A variants expressed by stably transfected mammalian cell lines. Cells (AM, THP-1, and CHO) were adhered to 96-well tissue culture plates and incubated with purified SP-A2 (1A^0^) and SP-A1 (6A^2^). Cells were then washed and lysed and the SP-A content of the lysates determined by Western dot blot and densitometry. A significant increase in binding of SP-A2 vs. SP-A1 (*p* = 0.0007) to AM was seen as was for the phagocytic THP-1 cells (*p* = 0.0015), but no differences were observed with the non-phagocytic CHO cells. **(B)** Depicts expression of SP-A binding proteins from AMs of hTG SP-A1 (6A^2^) and SP-A2 (1A^0^) mice. Bronchoalveolar lavage was performed on hTG SP-A2 (1A^0^) and SP-A1 (6A^2^) mice. AM were stained for SP-A binding proteins CD14 and TLR2 and analyzed by flow cytometry. A significant increase in expression of CD14 (*p* = 0.036) and TLR2 (*p* = 0.022) was seen in SP-A2 (1A^0^) AM vs. SP-A1 (6A^2^) AM. ^*^p < 0.05, ^***^p < 0.001.

Incubation of rat AMs with 2.5 μg/mL purified proteins SP-A1 (6A^2^), or SP-A2 (1A^0^) resulted in significant differences in binding between SP-A variants. SP-A2 (1A^0^) bound significantly more to AMs than SP-A1 (6A^2^) (Figure 8A). Similarly SP-A2 bound THP-1, a phagocytic cell line, significantly more than SP-A1 but no differences were observed when CHO cells, a non-phagocytic cell line was used (Figure 8A).

Next we investigated whether the AM differentially expresses cell surface proteins shown previously to bind SP-A. Expression of SP-A binding proteins such as CD14 and TLR2 on the cell surface of the AMs isolated from SP-A1 (6A^2^), or SP-A2 (1A^0^) mice were measured by flow cytometry. Figure 8B shows that the expression of these proteins differed significantly between the two variants. The SP-A2 (1A^0^) variant showed significantly higher expression of CD14 and TLR2 than SP-A1 (6A^2^). This differential expression of SP-A binding proteins on the cell surface of AM may play a role in the differential SP-A1 and SP-A2 binding.

## Discussion

Differences have been observed between SP-A1 and SP-A2 variants in their ability to enhance association of bacteria with the alveolar macrophage (AM), and SP-A2 variants were more effective than SP-A1 (18, 19, 40). A recent study indicated that the SP-A1 and SP-A2 variants differentially affect airway function in response to *K. pneumoniae* infection and methacholine challenge (23). In the current study, we wished to investigate whether SP-A variants differentially affect survival and whether rescue of SP-A KO mice with exogenous SP-A1 or SP-A2 improves survival. Toward this: we (a) Infected hTG mice carrying a different SP-A1 or SP-A2 variant or both SP-A1/SP-A2 (co-ex) as well as mice lacking SP-A (i.e., KO) with *K. pneumoniae* and studied their daily survival over a 14 day period; (b) Rescued *K. pneumoniae* infected KO mice with exogenous SP-A1 or SP-A2 treatment prior to and/or at the time infection; (c) Carried out pilot binding studies of SP-A1 (6A^2^) and SP-A2 (1A^0^) to the AM as well as expression of cell surface proteins from AMs of SP-A1 (6A^2^) and SP-A2 (1A^0^) mice. The results showed, (a) Gene-specific survival: co-ex = SP-A2 > SP-A1 > KO (male and female together); (b) Variant-specific survival co-ex (6A^2^/1A^0^) = 1A^0^ > 1A^3^ = 6A^2^ > 6A^4^ (male and female together); (c) Sex differences in survival in SP-A variants and KO mice, with females showing better survival than males; (d) The co-ex (6A^2^/1A^0^) did not exhibit sex differences; (e) exogenous treatment of KO mice with SPs (SP-A1 or SP-A2) proteins significantly improved the overall survival; (f) Differential gene-specific SP-A1 and SP-A2 binding was observed with phagocytic but not with non-phagocytic cells, as well as differential expression of cell surface proteins in AM from SP-A1 and SP-A2 mice. The latter may in part contribute to the observed differences.

The SP-A protein is a major component of innate immune system. It has been previously observed that SP-A KO mice are more susceptible to pneumonia and show poor survival compared to the wild type mice (4), and display enhanced susceptibility to pulmonary infections (41, 42). In the present study we observed significant SP-A1 and SP-A2 specific differences, with SP-A2 or co-ex having a better survival than SP-A1. The observed gene- specific differences in survival may be due to structural differences between SP-A1 and SP-A2. The sequence of human SP-A1 and SP-A2 genes differs within the coding region (10, 43). Four amino acid differences located within the collagen-like domain distinguish between SP-A1 and SP-A2 variants. These are Met66, Asp73, Ile81 and Cys85 for SP-A1, and Thr66, Asn73, Val81, and Arg85 for SP-A2. The amino acid at position 85 of the precursor molecule, where SP-A1 has a cysteine and SP-A2 has an arginine (10) is a key difference between SP-A1 and SP-A2. This single amino acid change has a major impact on SP-A oligomerization, lipopolysaccharide (LPS) aggregation, and phagocytosis (20). It was proposed that a cysteine in the collagen-like domain as it is in SP-A1 may cause micro instability resulting in a less stable protein (12). This change may play a key role in the gene-specific differences observed and may modulate functional capabilities mediated by the carbohydrate recognition domain (CRD) region. Thus, structural differences due to Cys85 and other amino acids may underlie the differences in function observed between SP-A1 and SP-A2.

Recently, SP-A1 and SP-A2 variants have been shown to play an important role in the differential outcome of airway function, with significant sex-, gene-, and variant-specific differences in lung function mechanics (23). In the current study, SP-A1 and SP-A2 variants also exhibited sex-, gene-, and variant- specific differences in survival. The 1A^3^ variant exhibited higher airway hyperreactivity compared to 1A^0^ for either sex, and the 6A^2^ and 6A^4^ variants exhibited diverse changes in both sexes in the lung function parameters studied (23). However, the 1A^3^ variant in the present study exhibited a decrease in survival than 1A^0^ in both sexes, and 6A^2^ and 6A^4^ variants did not differ in survival in either sex. The apparent discrepancies between SP-A2 variants in survival and lung function may be due to differences in the amount of time mice were exposed to *K. pneumoniae*, as lung functions were analyzed after 18 h of infection, whereas, the survival was monitored for a period of 14 days. We also speculate that the differences in amino acids at the collagen-like domain (as mentioned above) and in the CRD region of SP-A1 and SP-A2 at amino acid position 219 (Arg for 6A^2^ and Trp for 6A^4^) and at position 223 (Gln for 1A^0^ and Lys for 1A^3^) (10), may along with the lung microenvironment at the different time intervals further contribute to the apparent discrepancies in survival and lung function.

### Sex differences

In animal models, males exhibit a higher level of susceptibility to infection (*Candida albicans* and Mycobacterium marinum) (44, 45) and respiratory disease (Mycoplasma pulmonis) (46). Moreover, in clinical studies, sex influences significantly lung disease susceptibility, and males in general are more susceptible than females to lung disease, as observed in neonatal respiratory distress syndrome (RDS) after premature birth (47, 48), IPF, and COPD (49, 50), as well as different types of pneumonia (49, 51–53). This indicates that the relationship between sex and lung disease may be a complex one (50) and requires further investigation.

All SP-A1 and SP-A2 variants studied showed sex differences in survival with females showing a better rate of survival than males. This is consistent with our previous observations with animal models where wild type and SP-A KO mice showed similar sex differences after exposure to pneumonia and/or environmental stress (4, 36, 37). Sex hormones were shown to play a key role in the survival differences between males and females after infection and ozone-induced oxidative stress (38). In line with the sex differences observed in survival in the present study, are our recent findings where hTG SP-A1 and SP-A2 mice showed differences in their respiratory mechanics, with females exhibiting a significantly higher airway hyperreactivity in response to infection compared to males and the pattern was reversed after methacholine challenge (23). However, the SP-A1/SP-A2 (co-ex) mice showed no sex differences in survival, but did show sex differences in lung function mechanics with females to have higher airway hyperreactivity compared to males in response to infection. Interestingly, similar to survival, no sex differences were found in co-ex in response to infection and methacholine challenge (23). These differences (as mentioned above) may be due to (i) experimental differences in terms of the time interval of *K. pneumoniae* exposure, and (ii) the lung microenvironment at different time intervals after infection. In humans differences in the relative levels of SP-A1 and SP-A2 may further contribute to the observed differences in lung function mechanics and/or survival.

The hormonal mechanism as to how sex hormones regulate SP-A1 and SP-A2 are not known. However, other groups have reported that, SP-A gene expression in fetal lung tissues of various species to be regulated by a variety of hormones and factors, including retinoids, insulin, growth factors and cytokines, glucocorticoids and cAMP (54), estrogen related receptor –α, which is an important mediator of SP-A gene expression and its induction by cAMP (55), and testosterone to positively regulate the expression of SP-A (56). Moreover, human SP-A1 and SP-A2 genes are differentially regulated during development by cAMP and glucocorticoids (24, 25, 57).

E2 has been implicated in the immune response against infection, and involves the transcriptional activation of genes encoding TLR2 (58). SP-A modulates the lung inflammatory response by regulating macrophage TLR activity and specifically by enhancing the expression of TLR2 (59). It was observed that treatment of THP-1 cells (macrophage-like cell line) with E2 resulted in increased TLR2 mRNA and protein levels, indicating that the TLR2 is an estrogen-regulated gene whose expression is upregulated by interaction with ER-α (60). In our previous study, gonadectomy of FA-exposed females resulted in decreased survival, indicating that the female gonadal hormones (E2 and progesterone) are important for protection. Treatment of gonadectomized males with E2 increased the survival similar to intact females and survival of gonadectomized females treated with DHT was similar to that of intact males (38). Thus the available literature not only supports a role of sex hormones in animal survival but also sex hormones may affect expression of SP-A and TLR2 via which SP-A modulates lung inflammatory response.

We and others have shown that SP-A KO mice are more susceptible to pneumonia and show poor survival compared to the wild type mice (4), as well as display enhanced susceptibility to pulmonary infections (41, 42), with females being better than males indicating a role of SP-A in host defense. We have also shown that SP-A2 variants enhance bacterial cell association and phagocytosis more effectively than SP-A1 variants (18, 19), and these SP-A2 variants exhibited a better outcome of airway function (23). These together indicate a role of SP-A in infection and hat SP-A1 and SP-A2 differ in their activities in several lung processes/function and these may in part explain differences observed in the survival of SP-A1 and SP-A2 hTG mice.

### Rescue

A single or a double SP-A1 or SP-A2 treatment before and/or at the time of infection significantly improved survival. Also, a single SP-A treatment has been shown previously to nearly restore the AM proteomics profile of SP-A KO mice to that of wild type mice (61). Moreover, a single treatment with exogenous SP-A1 or SP-A2 differentially affected the AM proteome of SP-A KO mice (30). Further, the proteomic expression profile of the AM and the actin cytoskeleton of the AM from SP-A1 and SP-A2 mice are shown to differ (29, 31) as well as the AM miRNome is shown to differ in these mice (32). Together these indicate that the SP-A (SP-A1 and SP-A2) variants not only protect against common pathogens but point to future possibilities of use of SP-A proteins or perhaps peptides of SPs as a modulatory contributor to innate immune function.

### Binding and cell surface proteins

Phagocytosis of microspheres by a macrophage-like cell line, indicated that SP-A has a direct effect on the phagocytic cell (35). In the present study we observed that SP-A1 and SP-A2 differentially bind to AM, with SP-A2 binding being more than SP-A1. Similar differential binding was observed for a phagocytic cell line but not for a non-phagocytic cell line suggesting that cell surface molecules present on phagocytic cells mediate differential binding of SP-A1 and SP-A2. In fact in our pilot studies we observed in AMs from SP-A1 and SP-A2 hTG mice, a differential expression of cell surface proteins, CD14 and TLR2, previously shown to bind SP-A (62–64). In line with the above general possibility are our observations, where SP-A2 variants enhanced bacterial cell association and phagocytosis more effectively than SP-A1 variants (18, 19), and this activity was differentially compromised in response to oxidative stress (22). Also higher levels of pro-inflammatory cytokines have been observed in a macrophage-like cell line in response to SP-A2 treatment compared to SP-A1 treatment (13). Collectively these differential functional effects of SP-A1 and SP-A2 on the AM may, in part, contribute to the survival differences observed between SP-A1 and SP-A2 mice.

In summary, the genetics of the innate immunity and specifically the variants of human SP-A1 and SP-A2 affect survival after *K. pneumoniae* infection in a sex-, gene-, and variant-specific manner with females showing a better survival than males: (a) Gene-specific survival: co-ex = SP-A2 > SP-A1 > KO (male and female together); (b) Variant-specific survival co-ex (6A^2^/1A^0^) = 1A^0^ > 1A^3^ = 6A^2^ > 6A^4^ (male and female together); (c) Treatment with exogenous SP-A1 or SP-A2 before and/or at the time of infection improves survival significantly. We speculate that the differential binding of SP-A1 and SP-A2 to the AM mediated by differentially expressed cell surface proteins on the AM contributes to the observed differences. This study identifies a potential contributor (i.e., SP-A genetics) to differences in individual variability to lung disease susceptibility. Moreover, treatment with exogenous SP-A or SP-A mimics is likely to improve disease outcome in bacterial pneumonia or other types irritant-induced lung disorders.

## Ethics statement

All protocols used in this study were evaluated and approved by the Pennsylvania State University College of Medicine Institutional Animal Care and Use Committee and conformed to the guidelines of the National Institute of Health on the care and use of laboratory animals.

## Author contributions

NT: Performed experiments, ran statistics, analyzed and synthesized the data, contributed to the manuscript writing; TU: Performed binding assay, flow cytometry, mouse line maintenance, breeding, and infection; XZ: Performed mouse line maintenance, breeding, and infection; DP: Contributed to manuscript writing; JF: Designed the study and provided oversight to the entire project, involved in data analysis, integration, and writing of the manuscript. All authors read and approved the final manuscript.

### Conflict of interest statement

The authors declare that the research was conducted in the absence of any commercial or financial relationships that could be construed as a potential conflict of interest.

## Abbreviations

AM: Alveolar macrophages
ANOVA: Analysis of variance
SP-A: surfactant protein A
hSP-A: human SP-A
hTG: humanized transgenic
HBSS: Hank's Balanced Salt solution
K. pneumoniae: Klebsiella pneumoniae
KO: knockout
PBS: Phosphate Buffered Saline
SFTPA1: gene encoding SP-A1
SFTPA2: gene encoding SP-A2
TSB: Tryptic soy broth.
